# Behavioural and Structural Determinants of Mpox Vaccine Awareness and Uptake Among People With HIV in the Dominican Republic: A Cross‐Sectional Study

**DOI:** 10.1002/jia2.70128

**Published:** 2026-07-24

**Authors:** Robert Paulino‐Ramirez, Wilfredo R. Matias, Alexandre Chaumette, Hector Lora‐Rodríguez, Monica Thormann‐Peynado, Lucia Vargas

**Affiliations:** ^1^ Instituto De Medicina Tropical & Salud Global Universidad Iberoamericana (UNIBE) UNIBE Research Hub Santo Domingo Dominican Republic; ^2^ Division of Infectious Diseases Mass General Brigham Boston Massachusetts USA; ^3^ Department of Medicine Harvard Medical School Boston Massachusetts USA; ^4^ Department of Internal Medicine Yale School of Medicine New Haven Connecticut USA; ^5^ Ministry of Health Infectious Disease Division Santo Domingo Dominican Republic; ^6^ Ministry of Health Immune‐Preventable Division Santo Domingo Dominican Republic

**Keywords:** community engagement, Dominican Republic, mpox vaccination, people with HIV (PWH), structural barriers, vaccine uptake

## Abstract

**Introduction:**

Mpox disproportionately affects people with HIV (PWH), yet little is known about mpox vaccine awareness and uptake in low‐ and middle‐income countries. We evaluated mpox‐related knowledge, attitudes and practices (KAP) among PWH in the Dominican Republic to identify determinants of vaccine awareness and uptake.

**Methods:**

We conducted a cross‐sectional online survey of 98 PWH recruited through HIV clinics, community‐based organizations and social networks (January−May 2025). The questionnaire assessed sociodemographic characteristics, mpox‐related KAP, structural barriers and vaccination status. Univariable and multivariable logistic regression were used to examine factors associated with mpox vaccine awareness.

**Results:**

Half of the participants (49/98) were aware of the mpox vaccine. Individuals aged 31–40 years had significantly lower odds of awareness compared with those aged 21–30 years. Despite high levels of formal education, 79% rated their mpox knowledge as low, and most perceived minimal personal risk. Among participants aware of the vaccine, 71% had been vaccinated. Structural barriers, including inconvenient site locations and logistical challenges, were frequently reported, even among vaccinated individuals, indicating that access limitations rather than hesitancy were the dominant constraints. Trust in vaccinating organizations was high overall, and healthcare providers and community organizations were the primary sources of vaccine information. Peer and community influence emerged as notable facilitators of uptake, while lack of reliable information and concerns about vaccine safety contributed to hesitancy.

**Conclusions:**

Mpox vaccine awareness among PWH in the Dominican Republic was low, but willingness to vaccinate was high when individuals were informed and able to access services. Strengthening mpox preparedness will require expanding accessible vaccination sites, improving disease‐specific health literacy, and leveraging trusted community and clinical networks. Integrating mpox vaccination into routine HIV care delivery and enhancing community‐led outreach may substantially improve uptake in this and similar low‐middle income countries (LMIC) settings.

## 1 Introduction

Mpox has re‐emerged as a global public health threat, propelled by sustained human‐to‐human transmission. The 2022 multinational outbreak underscored the vulnerability of gay, bisexual and other men who have sex with men (GBMSM) and people with HIV (PWH) and led to the deployment of targeted vaccination campaigns across multiple regions. Despite early prioritization of PWH for immunization, studies from high‐income settings consistently show substantial gaps in vaccine awareness, risk perception and uptake, driven by intersecting sociodemographic, behavioural and structural determinants [1, 2, 3]. These gaps highlight limitations in current vaccine response strategies, particularly in low‐ and middle‐income countries where structural barriers and heterogeneous access to preventive services may amplify inequities among PWH.

Across diverse contexts, higher educational attainment, older age and identification as GBMSM have been associated with greater mpox knowledge and vaccine acceptance [4, 5, 6]. Perceptual factors, including risk perception and confidence in vaccine effectiveness, further shape willingness to vaccinate [7, 8]. While communication from trusted providers can improve knowledge, it does not always lead to increased vaccination rates, underscoring the complexity of vaccine‐related decision‐making in PWH [9]. Beyond individual perceptions, intersectional vulnerabilities—such as non‐White racial identity, low‐income or limited integration within men who have sex with men (MSM) social networks—further reduce awareness and access even in countries with universal healthcare [2, 4]. Collectively, these individual and structural barriers illustrate how disparities in information, trust and service availability constrain the ability of priority populations to translate knowledge into preventive action [2, 9, 10].

There remains a need to more precisely characterize the barriers and facilitators shaping mpox awareness and vaccine uptake across different geographic and sociocultural contexts. Cultural norms, religious beliefs and structural conditions influence how communities perceive mpox risk and vaccination, underscoring the importance of locally generated epidemiologic and behavioural data [1, 5, 7, 10]. Given its high HIV burden and concentrated epidemics among MSM and transgender women (TGW), the Dominican Republic (DR) represents a critical setting to explore the determinants of mpox knowledge and vaccine uptake among PWH [11]. To date, no studies have assessed mpox vaccine awareness or uptake in the Caribbean, or among PWH in Latin America.

In Latin America, mpox cases increased substantially during the 2022–2023 outbreak, with transmission concentrated among sexual and gender minority populations. In the DR, reported cases remained relatively low compared to larger regional epidemics; however, targeted vaccination efforts were implemented in 2023, focusing on PWH and other priority populations [12]. National vaccination strategies prioritized individuals at increased risk of exposure, including MSM and TGW, and were delivered through a combination of Ministry of Health facilities and community‐based organizations (CBOs). In the Americas, mpox transmission persists at relatively stable levels, with 801 cases reported in early 2026 and continued predominance of clade IIb, indicating ongoing endemic‐like transmission without a sustained decline [13]. Despite these efforts, publicly available data on vaccine coverage, uptake and access remain limited, underscoring the need for empirical studies examining awareness and barriers in this context. This study aimed to identify the sociodemographic, perceptual and structural determinants of mpox vaccine awareness and vaccination in a cohort of PWH in the DR, a setting characterized by high ART coverage but persistent inequalities in information access and healthcare infrastructure.

## 2 Methods

### 2.1 Study Design and Setting

We conducted a cross‐sectional survey to assess mpox vaccine awareness, attitudes and uptake among PWH in the DR. The study focused on MSM and TGW, populations prioritized during the national mpox vaccination effort and central to regional transmission dynamics. Recruitment occurred between January and May 2025, and participants were drawn from Santo Domingo and surrounding provinces.

### 2.2 Participants and Eligibility Criteria

Eligible participants were (1) aged 18 years or older, (2) had a confirmed HIV diagnosis through self‐report of a prior clinical diagnosis, (3) self‐identified as MSM or TGW, (4) resided in the DR and (5) provided electronic informed consent prior to participation. All participants had a confirmed HIV diagnosis and care prior to completing the survey as an eligibility criterion.

### 2.3 Recruitment and Sampling Strategy

We used a convenience sampling approach designed to reach PWH engaged in clinical care as well as those connected through community networks. Snowball sampling was implemented by inviting participants to share the survey link with peers within their social and community networks who met eligibility criteria. Therefore, the final sample reflects a mixture of participants: some were direct clients of clinics or CBOs, while others were recruited exclusively through peer referral or community social networks. This multi‐pronged strategy was designed to reduce reliance on institutional care settings and improve representation and engagement of PWH who are typically undeserved or not engaged in formal services. CBOs serving sexual and gender minority communities identified and enrolled eligible individuals with HIV through direct outreach and pre‐established trust networks. HIV specialty clinics in urban and semi‐urban areas shared the questionnaire with eligible participants during routine care visits. In the DR, HIV care is delivered through a combination of public health services and specialized clinics, with most clients receiving care through Ministry of Health‐supported programmes and affiliated institutions. Given the exploratory nature of this study and the absence of prior data on mpox vaccine awareness in this population, a formal sample size calculation was not performed. The final sample size reflects feasible recruitment within the study period and is consistent with similar knowledge, attitudes and practices (KAP) studies conducted in emerging infectious disease contexts. Recruitment sites partially overlapped with locations involved in the 2023–2024 vaccination campaign, particularly CBOs and urban HIV clinics, although not all recruitment sites directly provided vaccination services.

### 2.4 Data Collection and Measures

Data were collected using *Tangerine*, an open‐source, tablet/mobile‐based electronic data capture platform optimized for offline use in resource‐limited settings. Participants accessed the self‐administered questionnaire via a secure QR code, which directed them to the digital survey interface. The platform supported structured questionnaire deployment with embedded skip logic and validation rules. Data were stored securely under Universidad Iberoamericana (UNIBE) domain with password‐protected access. The survey was developed by the study team based on existing mpox vaccine literature and pre‐tested for clarity among community partners. We reviewed published KAP surveys from prior mpox outbreaks, as well as validated instruments used to assess risk perception, vaccine confidence and structural barriers to vaccination in HIV/STIs contexts [3, 4, 5, 6]. The survey captured sociodemographic and clinical characteristics, including age, gender identity, sexual orientation, educational attainment, employment status, residence, time since HIV diagnosis and viral suppression, and consisted primarily of structured, closed‐ended items with predefined response categories. These items included Likert‐scale and categorical response options evaluating perceived risk, concern about mpox, vaccine effectiveness, trust in institutions, and barriers such as site accessibility, logistical challenges and stigma. No open‐ended responses were collected for these domains. Items were adapted when appropriate to ensure alignment with the DR context, and new items were generated to address specific gaps. Structural barriers, including site accessibility, logistical challenges, scheduling difficulties and confidentiality concerns, were measured using items based on vaccine access frameworks for key populations. Mpox vaccine awareness was assessed through the question: “Before today, had you heard about the mpox vaccine?”(Yes/No). Participants additionally reported whether they were aware of the mpox vaccine, and whether they had received at least one dose.

Mpox‐related knowledge was assessed using a self‐reported item asking participants to rate their overall level of knowledge about mpox. Responses were categorized into three levels (low, moderate, high) based on predefined ordinal response options. For analytical purposes, these categories were retained as reported. Reasons for vaccine hesitancy and facilitators of vaccination were assessed using predefined, multiple‐response closed‐ended items. Participants were asked to select all applicable reasons (e.g. increased site availability, flexible hours, physician recommendation). These responses were analysed descriptively. No qualitative data collection or thematic analysis was conducted. All survey items were pilot tested for clarity and contextual relevance among community partners prior to implementation.

### 2.5 Statistical Analysis

Descriptive statistics were used to characterize the study population. Variables considered for inclusion in the multivariable logistic regression model were selected based on both conceptual relevance and empirical association. Age group, gender identity, educational attainment and residence were included a priori, given their established role as structural determinants of health information access and vaccine uptake. In addition, variables demonstrating a univariable association with mpox vaccine awareness at a threshold of *p* < 0.10 were considered for inclusion. For categorical variables, statistical significance in any category relative to the reference group warranted inclusion. Final model selection balanced statistical relevance and parsimony to avoid overfitting, given the sample size. Categorical variables were compared using chi‐square or Fisher's exact test as appropriate. Model diagnostics included assessment of multicollinearity and goodness‐of‐fit. Statistical analyses were performed using R version 4.3.2, with two‐sided *p*‐values <0.05 considered statistically significant [14].

### 2.6 Ethical Considerations

All recruitment and data collection procedures were reviewed and approved by the Universidad Iberoamericana (UNIBE) Institutional Review Board (CEI# 2024–39). The study was implemented by the DR research team, who conducted all recruitment and data collection. All participants provided electronic informed consent prior to participation and were assured of confidentiality and the voluntary nature of their involvement. Data were anonymized and securely stored in accordance with international research ethics standards involving vulnerable populations. After completion of data collection, a fully de‐identified dataset was shared with investigators at Mass General Brigham (MGB) for secondary analysis. The MGB Institutional Review Board determined that the analysis of de‐identified data qualified for exemption (ID# 3014). No identifiable information was accessed by U.S. investigators. All procedures adhered to the principles of the Declaration of Helsinki.

## 3 Results

### 3.1 Participant Characteristics

Ninety‐eight PWH in the DR completed the survey. Most were aged 31–40 years (58%), identified as cisgender men (91%) and identified as gay (77%). Most participants resided in the Santo Domingo metropolitan area (56%) (Table 1). Educational attainment (70% university‐level; 19% postgraduate) and employment was high, with 91% reporting either formal employment or self‐employment. All participants were receiving antiretroviral therapy, and 97% were virally suppressed; 60% had been diagnosed with HIV for ≥5 years. Missing data were minimal (<1%) and are not shown separately. Data on race/ethnicity and CD4 count were not collected.

**TABLE 1:** Demographic and clinical characteristics of participants (*N* = 98).

	*n* (%)
**Age group**	
21–30	20 (20%)
31–40	57 (58%)
≥41	21 (21%)
**Gender identity**	
Cis male	89 (91%)
Trans female	9 (9%)
**Sexual orientation**	
Gay	75 (77%)
Bisexual	15 (15%)
Heterosexual/other^a^	8 (8%)
**Education level**	
≤Secondary	10 (10%)
College/University	69 (70%)
Postgraduate	19 (19%)
**Employment status**	
Unemployed	8 (8%)
Self‐employed	22 (22%)
Formally employed	68 (69%)
**Residence**	
Santo Domingo Metro	55 (56%)
Other provinces^b^	43 (44%)
**Time since HIV diagnosis**	
≤2 years	11 (11%)
3–4 years	28 (29%)
≥5 years	59 (60%)
**Viral suppression** ^c^	95 (97%)

^a^Sexual orientation “Other” and “Heterosexual” merged into “Heterosexual/Other” for sparse cells.

^b^Residence collapsed to Santo Domingo Metro versus other provinces for cell size.

^c^All participants reported being on ART, antiretroviral therapy.

### 3.2 Mpox Vaccine Awareness

Mpox vaccine awareness was limited across the sample. Forty‐nine of 98 participants (50%) reported awareness of the vaccine (Table 2). In adjusted models, age group was the only variable significantly associated with awareness. Individuals aged 31–40 had significantly lower odds of awareness compared with those aged 21–30 (aOR 0.27; 95% CI 0.08−0.83). Awareness did not differ between those aged ≥41 and the youngest age group. TGW showed lower awareness in unadjusted analyses, although this association was not significant after adjustment. Individuals with higher formal educational attainment reported higher rates of mpox vaccine awareness in univariable analyses; however, this association was attenuated after adjustment. No significant associations were observed by sexual orientation, employment, residence or time since HIV diagnosis.

**TABLE 2:** Factors associated with mpox vaccine awareness among people living with HIV in the Dominican Republic (*N* = 98).

	Overall (*N* = 98)	Vaccine not aware (*n* = 49)	Vaccine aware (*n* = 49)	Univariable OR (95% CI)	*p*‐value	Adjusted OR^a^ (95% CI)	*p*‐value
**Age group**							
21–30	20	6 (30%)	14 (70%)	Ref	Ref	Ref	Ref
31–40	57	34 (60%)	23 (40%)	0.29 (0.09–0.83)	0.03	0.27 (0.08–0.83)	0.03
≥41	21	9 (43%)	12 (57%)	0.57 (0.15–2.05)	0.40	0.41 (0.09–1.70)	0.23
**Gender identity**							
Cis male	89	41 (46%)	48 (54%)	Ref	Ref	Ref	Ref
Trans female	9	8 (89%)	1 (11%)	0.11 (0.01–0.62)	0.04	0.07 (0.00–1.54)	0.12
**Sexual orientation**							
Gay	75	37 (49%)	38 (51%)	Ref	Ref	—	—
Bisexual	15	6 (40%)	9 (60%)	1.46 (0.48–4.74)	0.50	—	—
Heterosexual/other	8	6 (75%)	2 (25%)	0.32 (0.05–1.51)	0.19	—	—
**Education**							
≤Secondary	10	8 (80%)	2 (20%)	Ref	Ref	Ref	Ref
College/university	69	34 (49%)	35 (51%)	4.12 (0.95–28.6)	0.09	0.55 (0.02–9.28)	0.67
Postgraduate	19	7 (37%)	12 (63%)	6.86 (1.28–54.8)	0.04	0.95 (0.03–19.3)	0.98
**Employment status**							
Unemployed	8	6 (75%)	2 (25%)	Ref	Ref	—	—
Self‐employed	22	11 (50%)	11 (50%)	3.00 (0.55–23.7)	0.20	—	—
Formally employed	68	32 (47%)	36 (53%)	3.37 (0.72–24.2)	0.15	—	—
**Residence**							
Santo Domingo Metro	55	27 (49%)	28 (51%)	Ref	Ref	—	—
Other provinces	43	22 (51%)	21 (49%)	0.92 (0.41–2.05)	0.84	0.74 (0.30–1.78)	0.51
**Time since HIV diagnosis**							
≤2 years	11	5 (45%)	6 (55%)	Ref	Ref	–	—
3–4 years	28	13 (46%)	15 (54%)	0.96 (0.23–3.93)	0.60	—	—
≥5 years	59	31 (53%)	28 (47%)	0.75 (0.20–2.76)	0.67	—	—

Abbreviations: CI, confidence interval; OR, odds ratio; Ref, reference category.

^a^Multivariable model adjusted for age group, gender identity, education and residence.

### 3.3 Vaccination Uptake Among Participants Aware of the Vaccine

Vaccination uptake among the 49 participants aware of the vaccine was high: 35 (71%) had received at least one dose (Table 3). Uptake did not differ meaningfully across age groups, gender identities and sexual orientation. When examined within HIV‐diagnosis duration categories, 23 of 28 participants diagnosed ≥5 years earlier had been vaccinated (82%); however, this comparison should be interpreted descriptively because of small cell counts. Participants were asked whether they had received at least one dose of the mpox vaccine; information on the number of doses or completion of the vaccination schedule was not collected.

**TABLE 3:** Characteristics of participants aware of the mpox vaccine, by vaccination status (*N* = 49).

	Overall *N* (%)	Not vaccinated (*n* = 14) *n* (%)	Vaccinated (*n* = 35) *n* (%)	*p*‐value
**Age group**				0.97
21–30	14 (28.6)	4 (28.6)	10 (28.6)	
31–40	23 (46.9)	7 (50)	16 (45.7)	
≥41	12 (24.5)	3 (21.4)	9 (25.7)	
**Gender identity**				1.00*
Cis male	48 (98.0)	14 (100)	34 (97.1)	
Trans female	1 (2.0)	0 (0)	1 (2.9)	
**Sexual orientation**				0.66*
Gay	38 (77.6)	11 (78.6)	27 (77.1)	
Bisexual	9 (18.4)	2 (14.3)	7 (20.0)	
Heterosexual/other	2 (4.1)	1 (7.1)	1 (2.9)	
**Education**				0.79*
≤Secondary	2 (4.1)	0 (0)	2 (5.7)	
College/university	35 (71.4)	10 (71.4)	25 (71.4)	
Postgraduate	12 (24.5)	4 (28.6)	8 (22.9)	
**Employment status**				0.85*
Unemployed	2 (4.1)	0 (0)	2 (5.7)	
Self‐employed	11 (22.4)	4 (28.6)	7 (20.0)	
Formally employed	36 (73.5)	10 (71.4)	26 (74.3)	
**Residence**				0.58
Santo Domingo Metro	28 (57.1)	7 (50.0)	21 (60.0)	
Other provinces	21 (42.9)	7 (50.0)	14 (40.0)	
**Time since HIV diagnosis**				0.11
≤2 years	6 (12.2)	3 (21.4)	3 (8.6)	
3–4 years	15 (30.6)	6 (42.9)	9 (25.7)	
≥5 years	28 (57.1)	5 (35.7)	23 (65.7)	

*Note*: Data are presented as *n* (column %) unless otherwise specified. Percentages in the “Not vaccinated” and “Vaccinated” columns are calculated using column totals. *P*‐values were calculated using the chi‐square (*x* ^2^) test or Fisher's exact test* when expected cell counts were <5. Missing data were minimal (<1%) and excluded from analyses.

### 3.4 Knowledge, Attitudes and Perceptions by Awareness Status

Across the full sample, self‐rated mpox knowledge was low (79% reporting low knowledge), particularly among participants unaware of the vaccine (96%) (Table 4). Most participants perceived low personal risk (83%) and expressed low concern about mpox (90%), although those aware of the vaccine reported slightly higher perceived risk and concern. Beliefs about vaccine effectiveness were generally favourable (rated as high by 61% of participants) and more positive among the vaccine‐aware group (69% vs. 53%). Trust in vaccinating organizations was high overall (68%) and especially high among those aware of the vaccine (96% reporting moderate‐to‐high combined). Preference to delay vaccination until more information became available (56%) and concern about adverse events related to HIV status (59%) were common and did not differ substantially by awareness.

**TABLE 4:** Mpox vaccine‐related knowledge, attitudes and practices (KAP) by mpox vaccine awareness.

Characteristic	Overall^a^ *N* = 98 (%)	Vaccine unaware *n* = 49 *n* (%)	Vaccine aware *n* = 49 *n* (%)
**Self‐rated mpox knowledge**			
Low	77 (79%)	47 (96%)	30 (61%)
Mid	12 (12%)	2 (4.1%)	10 (20%)
High	9 (9.2%)	0 (0%)	9 (18%)
**Perceived personal risk**			
Low	81 (83%)	45 (92%)	36 (73%)
Mid	10 (10%)	2 (4.1%)	8 (16%)
High	7 (7.1%)	2 (4.1%)	5 (10%)
**Concern about mpox**			
Low	88 (90%)	46 (94%)	42 (86%)
Mid	6 (6.1%)	1 (2.0%)	5 (10%)
High	4 (4.1%)	2 (4.1%)	2 (4.1%)
**Perceived vaccine effectiveness**			
Low	23 (23%)	17 (35%)	6 (12%)
Mid	15 (15%)	6 (12%)	9 (18%)
High	60 (61%)	26 (53%)	34 (69%)
**Trust in vaccinating organizations**			
Low	14 (14%)	12 (24%)	2 (4.1%)
Mid	17 (17%)	4 (8.2%)	13 (27%)
High	67 (68%)	33 (67%)	34 (69%)
**Prefers to wait for more information before vaccinating**			
Low	24 (24%)	12 (24%)	12 (24%)
Mid	19 (19%)	9 (18%)	10 (20%)
High	55 (56%)	28 (57%)	27 (55%)
**Concern about AEs due to HIV**			
Low	23 (23%)	12 (24%)	11 (22%)
Mid	17 (17%)	7 (14%)	10 (20%)
High	58 (59%)	30 (61%)	28 (57%)

Abbreviations: AEs, adverse events; KAP, knowledge, attitudes and practices.

^a^Percentages are column‐wise within groups. Overall column shows totals across groups.

### 3.5 KAP and Structural Barriers Among Vaccine‐Aware Participants

Among individuals aware of the vaccine, vaccinated and unvaccinated participants reported similarly low mpox knowledge, perceived risk and concern (Table 5). Vaccinated participants more frequently reported high trust in vaccinating organizations than unvaccinated participants (74% vs. 57%). Vaccinated participants were less likely to report high concern about HIV‐related adverse events than unvaccinated participants (46% vs. 86%). Healthcare providers were the most common information source (47%), followed by community organizations (22%), with social media playing a smaller role (12%). Structural barriers were widespread: 78% of participants reported that vaccination sites were not conveniently located, and 69% experienced logistical difficulties. Notably, most vaccinated participants (83%) still reported logistical barriers, underscoring that access limitations—not hesitancy—were the primary obstacles to vaccine uptake. Peer and community influence differed by vaccination status, with vaccinated participants more frequently reporting encouragement from peer leaders and community organizations.

**TABLE 5:** Mpox vaccine‐related knowledge, attitudes, practices (KAP) and barriers among participants aware of the vaccine, stratified by vaccination status.

Characteristic	Overall^a^ *N* = 49	Unvaccinated *n* = 14	Vaccinated *n* = 35
**Self‐rated mpox knowledge**			
Low	30 (61%)	6 (43%)	24 (69%)
Mid	10 (20%)	3 (21%)	7 (20%)
High	9 (18%)	5 (36%)	4 (11%)
**Perceived personal risk**			
Low	36 (73%)	8 (57%)	28 (80%)
Mid	8 (16%)	3 (21%)	5 (14%)
High	5 (10%)	3 (21%)	2 (5.7%)
**Concern about mpox**			
Low	42 (86%)	10 (71%)	32 (91%)
Mid	5 (10%)	3 (21%)	2 (5.7%)
High	2 (4.1%)	1 (7.1%)	1 (2.9%)
**Perceived vaccine effectiveness**			
Low	6 (12%)	1 (7.1%)	5 (14%)
Mid	9 (18%)	2 (14%)	7 (20%)
High	34 (69%)	11 (79%)	23 (66%)
**Trust in vaccinating orgs**			
Low	2 (4.1%)	1 (7.1%)	1 (2.9%)
Mid	13 (27%)	5 (36%)	8 (23%)
High	34 (69%)	8 (57%)	26 (74%)
**Prefers to wait for more information before vaccinating**			
Low	12 (24%)	0 (0%)	12 (34%)
Mid	10 (20%)	4 (29%)	6 (17%)
High	27 (55%)	10 (71%)	17 (49%)
**Level of concern about AEs due to HIV**			
Low	11 (22%)	0 (0%)	11 (31%)
Mid	10 (20%)	2 (14%)	8 (23%)
High	28 (57%)	12 (86%)	16 (46%)
**Source of vaccine information**			
Community organization	11 (22%)	2 (14%)	9 (26%)
Health provider	23 (47%)	6 (43%)	17 (49%)
Social media	6 (12%)	3 (21%)	3 (8.6%)
Dating apps	0 (0%)	0 (0%)	0 (0%)
Mass media	9 (18%)	3 (21%)	6 (17%)
Other	0 (0%)	0 (0%)	0 (0%)
**Vaccination site convenient**			
No	38 (78%)	11 (79%)	27 (77%)
Yes	11 (22%)	3 (21%)	8 (23%)
**Logistical challenges**			
No	15 (31%)	9 (64%)	6 (17%)
Yes	34 (69%)	5 (36%)	29 (83%)
**Stigma as a barrier**			
No	40 (82%)	11 (79%)	29 (83%)
Yes	9 (18%)	3 (21%)	6 (17%)
**Peer/community leader influence**			
No	29 (59%)	13 (93%)	16 (46%)
Maybe	4 (8.2%)	1 (7.1%)	3 (8.6%)
Yes	16 (33%)	0 (0%)	16 (46%)
**Community org encouragement**			
No	29 (59%)	11 (79%)	18 (51%)
Maybe	5 (10%)	1 (7.1%)	4 (11%)
Yes	15 (31%)	2 (14%)	13 (37%)
**Reason that influenced vaccine hesitancy (if any)**			
Safety concerns	15 (31%)	3 (21%)	12 (34%)
Effectiveness doubts	19 (39%)	4 (29%)	15 (43%)
Lack of reliable information	30 (61%)	6 (43%)	24 (69%)
Personal/religious beliefs	2 (4.1%)	0 (0%)	2 (5.7%)
Low perceived risk	31 (63%)	5 (36%)	26 (74%)
Other reason	1 (2.0%)	1 (7.1%)	0 (0%)
**What would make it easier for you to get vaccinated?**			
More sites available	48 (98%)	14 (100%)	34 (97%)
Flexible hours	39 (80%)	8 (57%)	31 (89%)
No appointments	16 (33%)	5 (36%)	11 (31%)
No personal info required	8 (16%)	3 (21%)	5 (14%)
No consent/forms	3 (6.1%)	2 (14%)	1 (2.9%)
**Which strategies would increase mpox vaccine acceptance?**			
Social media campaigns	43 (88%)	11 (79%)	32 (91%)
Physician/expert‐led campaigns	20 (41%)	7 (50%)	13 (37%)
Community talks	11 (22%)	3 (21%)	8 (23%)
More accessible sites	32 (65%)	8 (57%)	24 (69%)
Confidentiality guarantees	5 (10%)	5 (36%)	0 (0%)
Other strategies	1 (2.0%)	1 (7.1%)	0 (0%)
**Most helpful type of support to encourage vaccination**			
Personalized information	15 (31%)	3 (21%)	12 (34%)
Testimonials (PWH)	5 (10%)	2 (14%)	3 (8.6%)
Community leaders’ involvement	8 (16%)	4 (29%)	4 (11%)
Active provider recommendations	20 (41%)	4 (29%)	16 (46%)
Other	1 (2.0%)	1 (7.1%)	0 (0%)

Abbreviations: AEs, adverse events; KAP, knowledge, attitudes and practices; PWH, people living with HIV.

^a^Percentages are column‐wise within vaccination groups among participants aware of the vaccine.

### 3.6 Reasons for Hesitancy and Facilitators of Vaccination

Among the vaccine‐aware group, reasons for hesitancy included low perceived risk (63%), lack of reliable information (61%), doubts about vaccine effectiveness (39%) and safety concerns related to HIV (31%). Nearly all participants indicated that increasing the number of vaccination sites (98%) and offering flexible hours (80%) would facilitate vaccination. Social media campaigns (88%), improved site accessibility (65%), and physician‐led messaging (41%) were cited as promising strategies, with provider recommendation (41%) and personalized information (31%) identified as the most helpful supports.

## 4 Discussion

In this cross‐sectional study of PWH in the DR, mpox vaccine awareness was low, yet vaccination uptake among those aware of the vaccine was high. Most participants reported low mpox knowledge and low perceived personal risk, and structural barriers—rather than attitudinal hesitancy—emerged as the dominant obstacles to vaccination. To our knowledge, this is the first study to characterize mpox vaccine awareness and uptake among PWH in the Caribbean, a region where empirical data on mpox prevention remains scarce despite increasing vulnerability.

Our findings align with data from Brazil, where mpox vaccine hesitancy among MSM has been primarily driven by low perceived risk, limited access to reliable information and concerns about vaccine safety, suggesting that information and perceptual barriers may coexist with, but differ from, the structural access constraints observed in our settings [16]. Similar patterns have been observed in Colombia, where moderate knowledge of mpox (64%) coexists with high willingness to vaccinate (89.5%) but limited behavioural change, highlighting the persistence of informational gaps and risk misperception across the region, even as structural access barriers appear to play a more dominant role in our setting [17].

Mpox vaccine awareness was limited to half of the cohort and was lowest among individuals aged 31–40 years. This pattern contrasts with data from high‐income settings, where older age has been associated with greater mpox awareness and uptake [4, 5, 6]. Younger PWH may also be more embedded within social and sexual networks where mpox information circulated rapidly during the 2022 outbreak. Younger PWH may also have more frequent interactions with community organizations or HIV clinics, which were the primary sources of information in this study. Although TGW demonstrated lower awareness in unadjusted analyses, the sample size was limited; the attenuation of this association in multivariable models may reflect limited statistical power due to the sample or confounding by socioeconomic and structural factors. Nevertheless, this finding aligns with documented disparities in access to sexual health information for gender minority populations. Higher formal educational attainment was associated with greater mpox vaccine awareness in univariable analyses, consistent with global evidence linking education to preventive health‐seeking behaviours and literacy [15, 16, 17].

However, despite the high proportion of participants with higher education, 79% reported low mpox‐related knowledge, suggesting a gap between general educational attainment and disease‐specific health literacy. This discrepancy indicates that public health messaging on mpox may not have effectively reached key populations during the outbreak. Consistent with findings from other settings, lack of reliable information and low perceived susceptibility were the most frequently reported contributors to vaccine hesitancy [16, 17]. This suggests that formal education alone may not be sufficient to ensure adequate disease‐specific health literacy in the context of emerging infectious threats. Vaccinated participants expressed fewer concerns about HIV‐related adverse events than unvaccinated individuals, suggesting that clear messaging on mpox vaccine safety for PWH may be an important but underutilized communication strategy.

Structural barriers to vaccination were common. Most vaccine‐aware participants reported that vaccination sites were not conveniently located and that logistical constraints, including distance, limited hours and appointment requirements, impeded access. Notably, most vaccinated individuals still reported substantial logistical challenges, indicating that vaccination often occurred despite meaningful constraints, and that structural access issues—rather than hesitancy—may be the primary limiting factor to uptake. These findings highlight gaps in service integration and underscore the importance of embedding mpox vaccination within routine HIV care delivery platforms where PWH are already engaged, and trust is well‐established to mitigate access constraints.

Social and community influences emerged as important facilitators of vaccination. Encouragement from peer and community leaders was reported exclusively by vaccinated participants, underscoring the critical role of trusted networks in converting awareness into action. This mirrors decades of evidence from HIV prevention showing that peer‐led and community‐based approaches substantially improve uptake of new prevention technologies among sexual and gender minority populations. Participants also endorsed social media outreach, physician‐led education and personalized information as strategies to increase acceptance, emphasizing the importance of multimodal and culturally tailored communication.

Taken together, these findings point towards actionable strategies: integrating mpox vaccination into HIV care workflows, expanding geographically accessible vaccination sites, investing in targeted communication addressing PWH‐specific safety concerns and partnering with trusted community organizations to enhance outreach. These patterns align with global priorities for equitable vaccine delivery and reinforce the need for implementation strategies that address structural and community‐level determinants of mpox prevention in LMIC settings.

Few studies have closely examined KAP related to mpox vaccination among PWH in low‐ and middle‐income countries such as the DR. Our findings reveal a disconnect between strong engagement in HIV care and low levels of mpox‐specific awareness, and they illustrate how structural barriers—particularly limited site accessibility—can undermine vaccination even among motivated individuals. These insights can inform targeted preparedness strategies in the Caribbean and similar LMIC contexts.

This study has several limitations. First, the relatively small sample size may limit the external validity of our findings and the ability to detect more subtle associations. Second, reliance on self‐reported measures, which may not fully reflect actual risk perceptions or behaviours and the cross‐sectional design, which precludes causal inference. Third, the study population was characterized by relatively high levels of educational attainment and employment compared to the general population of PWH in the DR. This likely reflects, in part, the recruitment strategy, which relied on engagement through clinics, CBOs and digital networks. As a result, individuals with lower socioeconomic status, limited access to care or weaker social network integration may be underrepresented. This is particularly relevant given that educational attainment was associated with mpox vaccine awareness in univariable analyses, suggesting that our findings may overestimate awareness levels relative to more socioeconomically disadvantaged populations. Fourth, the absence of participants aged 18–21 years limits the generalizability of our findings to younger individuals, a group that may have distinct patterns of risk perception, health‐seeking behaviour and vaccine uptake. This also limited precision for subgroup analyses, particularly among TGW and participants living in rural settings. Fifth, as with all cross‐sectional surveys, the data are subject to self‐report and potential social desirability bias, which may have influenced responses related to sensitive behaviours and perceptions. Nonetheless, the study provides timely and context‐specific evidence on mpox vaccine dynamics among PWH in the DR and contributes to a sparse evidence base from low‐ and middle‐income settings.

## 5 Conclusions

This study provides the first evidence on mpox vaccine awareness and uptake among PWH in a Caribbean setting. With only half of participants aware of the mpox vaccine and 71% uptake among those aware, our findings indicate that structural and informational barriers—rather than hesitancy—shape vaccination dynamics in this population. Efforts to enhance mpox preparedness should prioritize improving disease‐specific health literacy, reducing structural access barriers, and leveraging trusted community and clinical networks. Integrating mpox vaccination into routine HIV care delivery and strengthening community‐led communication channels may substantially improve access and uptake. These insights can guide mpox preparedness strategies in the DR and inform broader implementation efforts across Latin America and other LMIC settings.

## Author Contributions

RPR and WRM contributed equally to this work and share first authorship. RPR conceptualized and designed the study, developed the methodology, supervised data collection, interpreted the data, and co‐led manuscript drafting and critical revision. WRM conducted data analysis and interpretation and co‐led manuscript drafting and critical revision. AC contributed to data interpretation and manuscript drafting. HLR assisted with data management and contributed to data interpretation. MTP and LV, representing the DR Ministry of Health, coordinated the national mpox vaccination campaign and provided programmatic insights and critical revisions to the manuscript. All authors reviewed and approved the final manuscript.

## Funding

WRM was supported by the US National Institute of Allergy and Infectious Diseases (T32 AI007433) and the Harvard University Center for AIDS Research (CFAR), an NIH‐funded programme (P30 AI060354). The funders had no role in study design, data collection, analysis, decision to publish or preparation of the manuscript.

## Conflicts of Interest

The authors declare no conflicts of interest.

## Data Availability

De‐identified data supporting the findings of this study are available from the corresponding author upon reasonable request and in accordance with institutional IRB requirements.

## References

[1] L. Fu , Y. Sun , Y. Li , et al., “Perception of and Vaccine Readiness Towards Mpox Among Men Who Have Sex With Men Living With HIV in China: A Cross‐Sectional Study,” Vaccines 11, no. 3 (2023): 528. 36992114 10.3390/vaccines11030528 PMC10059946

[2] N. Alavian , A. Mourad , E. W. Woodhouse , et al., “Disparities in Mpox Vaccination Among Priority Populations During the 2022 Outbreak,” Open Forum Infectious Diseases 10, no. 9 (2023): ofad434. 37662451 10.1093/ofid/ofad434 PMC10472485

[3] D. Zucman , E. Fourn , P. Touche , C. Majerholc , and A. Vallée , “Monkeypox Vaccine Hesitancy in French Men Having Sex With Men With PrEP or Living With HIV in France,” Vaccines 10, no. 10 (2022): 1629. 36298494 10.3390/vaccines10101629 PMC9609833

[4] S. Paparini , R. Whitacre , M. Smuk , et al., “Public Understanding and Awareness of and Response to Monkeypox Virus Outbreak: A Cross‐Sectional Survey of the Most Affected Communities in the United Kingdom During the 2022 Public Health Emergency,” HIV Medicine 24, no. 5 (2023): 544–557. 36385726 10.1111/hiv.13430

[5] T. S. Torres , M. S. T. Silva , C. Coutinho , et al., “Evaluation of Mpox Knowledge, Stigma, and Willingness to Vaccinate for Mpox: Cross‐Sectional Web‐Based Survey Among Sexual and Gender Minorities,” JMIR Public Health and Surveillance 9 (2023): e46489. 37459174 10.2196/46489 PMC10411424

[6] E. W. Andersen , P. Kulie , A. D. Castel , et al., “Mpox Awareness, Risk Reduction, and Vaccine Acceptance Among People With HIV in Washington, DC,” Pathology 13, no. 2 (2024): 124. 10.3390/pathogens13020124 PMC10891655 38392862

[7] Z. Iliyasu , A. A. Kwaku , N. S. Nass , et al., “Risk Perception and mpox Vaccine Acceptability Among People Living With HIV in Northern Nigeria,” Transactions of the Royal Society of Tropical Medicine and Hygiene 119, no. 5 (2025): 487–497. 39731207 10.1093/trstmh/trae135 PMC12050367

[8] N. S. Wong , B. C. K. Wong , M. P. Lee , et al., “Mpox Vaccination for Men Who Have Sex With Men and Their Differential Risk of Exposure and Infection,” Human Vaccines & Immunotherapeutics 19, no. 2 (2023): 2252263. 37649367 10.1080/21645515.2023.2252263 PMC10472867

[9] V. Crosato , B. Formenti , M. Gulletta , et al., “Perception and Awareness About Monkeypox and Vaccination Acceptance in an at‐Risk Population in Brescia, Italy: An Investigative Survey,” AIDS and Behavior 28, no. 5 (2024): 1594–1600. 38240947 10.1007/s10461-024-04271-9 PMC11069468

[10] A. S. W. Svartstein , A. D. Knudsen , S. L. Heidari , et al., “Mpox Incidence and Vaccine Uptake in Men Who Have Sex With Men and Are Living With HIV in Denmark,” Vaccines 11, no. 7 (2023): 1167. 37514983 10.3390/vaccines11071167 PMC10385255

[11] P. Rojas , R. Malow , B. Ruffin , E. M. Rothe , and R. Rosenberg , “The HIV/AIDS Epidemic in the Dominican Republic: Key Contributing Factors,” Journal of the International Association of Providers of AIDS Care 10, no. 5 (2011): 306–315. 10.1177/1545109710397770 PMC5637397 21368008

[12] R. Paulino‐Ramirez , W. R. Matias , H. Lora‐Rodríguez , et al., “Clinical and Epidemiologic Characteristics of Mpox Cases, Dominican Republic, July 2022–February 2023,” Emerging Infectious Diseases 31, no. 5 (2025): 1060–1062, https://wwwnc.cdc.gov/eid/article/31/5/24‐1299_article. 40096741 10.3201/eid3105.241299 PMC12044227

[13] Pan American Health Organization/World Health Organization , Epidemiological Update: Mpox in the Americas Region, 23 April 2026 Available from: (PAHO/WHO, 2026) [cited 2026 Apr 28]. https://www.paho.org.

[14] R Core Team , R: A Language and Environment for Statistical Computing [Internet] Available from: (R Foundation for Statistical Computing, 2023) [cited 2025 Nov 18]. https://www.R‐project.org/.

[15] T. F. Anagaw , E. Melaku Mazengia , E. K. Bogale , et al., “Health‐Seeking Behavior Among Non‐Communicable Disease Patients Globally, Systematic Review and Meta‐Analysis,” SAGE Open Medicine 11 (2023). 10.1177/20503121231215236 PMC10702414 38078206

[16] G. R. S. Santos , C. J. N. Ribeiro , J. F. C. Santos Júnior , et al., “Mpox Vaccine Hesitancy Among Brazilian Men Who Have Sex With Men: A National Cross‐Sectional Study,” Vaccines (Basel) 12, no. 11 (2024): 1229, 10.3390/vaccines12111229. 39591132 PMC11598715

[17] C. Díaz‐Brochero , M. Barriga , J. F. Ramirez , et al., “Mpox Knowledge, Risk Perception, Attitudes and Willingness to Vaccinate in Colombia's LGBTIQ+ Communities: Online Survey (CoSex),” Travel Medicine and Infectious Disease 65 (2025): 102848, 10.1016/j.tmaid.2025.102848. 40158767

